# The Role of Mitochondria-Linked Fatty-Acid Uptake-Driven Adipogenesis in Graves Orbitopathy

**DOI:** 10.1210/endocr/bqab188

**Published:** 2021-09-02

**Authors:** Lei Zhang, Pavandeep Rai, Satomi Miwa, Mohd Shazli Draman, D Aled Rees, Anjana S Haridas, Daniel S Morris, Andrew R Tee, Marian Ludgate, Doug M Turnbull, Colin M Dayan

**Affiliations:** 1 School of Medicine, Cardiff University, Heath Park Hospital, Cardiff, CF14 4XN, UK; 2 Department of Ophthalmology, Cardiff & Vale University Health Board, Heath Park Hospital , Cardiff CF14 4XW, UK; 3 Wellcome Centre for Mitochondrial Research, Translational and Clinical Research Institute, Faculty of Medical Sciences, Newcastle University, Newcastle, NE2 4HH, UK; 4 Biosciences Institute, Newcastle University, Newcastle Upon Tyne, NE4 5PL, UK

**Keywords:** Graves orbitopathy, orbital adipose tissue, fatty acid uptake, adipogenesis, mitochondria OXPHOS, ATP production

## Abstract

**Context:**

Depot-specific expansion of orbital adipose tissue (OAT) in Graves orbitopathy (GO; an autoimmune condition producing proptosis, visual impairment and reduced quality of life) is associated with fatty acid (FA)-uptake–driven adipogenesis in preadipocytes/fibroblasts (PFs).

**Objective:**

This work sought a role for mitochondria in OAT adipogenesis in GO.

**Methods:**

Confluent PFs from healthy OAT (OAT-H), OAT from GO (OAT-GO) and white adipose tissue in culture medium compared with culture medium containing a mixed hormonal cocktail as adipogenic medium (ADM), or culture-medium containing FA-supplementation, oleate:palmitate:linoleate (45:30:25%) with/without different concentration of mitochondrial biosubstrate adenosine 5′-diphosphate/guanosine 5′-diphosphate (ADP/GDP), AICAR (adenosine analogue), or inhibitor oligomycin-A for 17 days. Main outcome measures included oil-red-O staining and foci count of differentiated adipocytes for in vitro adipogenesis, flow cytometry, relative quantitative polymerase chain reaction, MTS-assay/10^6^ cells, total cellular-ATP detection kit, and Seahorse-XFe96-Analyzer for mitochondria and oxidative-phosphorylation (OXPHOS)/glycolysis-ATP production analysis.

**Results:**

During early adipogenesis before adipocyte formation (days 0, 4, and7), we observed OAT-specific cellular ATP production via mitochondrial OXPHOS in PFs both from OAT-H and OAT-GO, and substantially disrupted OXPHOS-ATP/glycolysis-ATP production in PFs from OAT-GO, for example, a 40% reduction in OXPHOS-ATP and trend-increased glycolysis-ATP production on days 4 and 7 compared with day 0, which contrasted with the stable levels in OAT-H. FA supplementation in culture-medium triggered adipogenesis in PFs both from OAT-H and OAT-GO, which was substantially enhanced by 1-mM GDP reaching 7% to 18% of ADM adipogenesis. The FA-uptake–driven adipogenesis was diminished by oligomycin-A but unaffected by treatment with ADP or AICAR. Furthermore, we observed a significant positive correlation between FA-uptake–driven adipogenesis by GDP and the ratios of OXPHOS-ATP/glycolysis-ATP through adipogenesis of PFs from OAT-GO.

**Conclusion:**

Our study confirmed that FA uptake can drive OAT adipogenesis and revealed a fundamental role for mitochondria-OXPHOS in GO development, which provides potential for therapeutic interventions.

Graves orbitopathy (GO), also called thyroid eye disease, is a disfiguring disease of the orbit with a higher incidence in women (80%) (1, 2). The uncontrolled expansion of orbital adipose tissue (OAT) contributes to proptosis, double vision, and in some cases visual loss. GO develops mainly in the context of an autoimmune condition, Graves disease, in which thyrotropin receptor (*TSHR*) activation by thyroid-stimulating antibodies mimics the action of TSH, producing hyperthyroidism (1, 2). The TSHR is also detected and increased in OAT in GO, and is an essential cellular target both for GO and Graves disease (3-6). Previous GO studies have examined the crosstalk of signaling pathways via 2 cell-surface receptors, *TSHR* and insulin-like growth factor 1 receptor (*IGF1R*), focusing on disease-targeted preadipocytes/fibroblasts (PFs) embedded in OAT (7, 8).

OAT-PFs are mesenchymal stem cell (MSCs) with multidifferentiation potentials, as has been described by ourselves and others (9-11). In GO patients, the excessive adipogenesis via lineage-specific differentiation of PFs in OAT occurs rapidly (1, 12). By contrast, WAT (white adipose tissues) from the same individual typically shrinks in Graves disease because of hyperthyroidism (2). Previously we described a cell-specific signaling network (*PI3K*/*Akt*/*mTORC1*/*FOXOs*) in PFs from human OAT not present in WAT (13, 14). The identified pathways interact with *TSHR*/*IGF1R* signaling to play essential roles in the depot-specific OAT expansion in GO (2). Our recent work has demonstrated that OAT is a distinctive metabolic-quiescent fat depot that neither stores additional triglycerides (TAGs) in obesity (15) nor burns fatty acids (FAs) (11), in contrast to WAT and BAT (brown adipose tissue)/BRITE (BRown in whITE), respectively. OAT also displays a unique FA-uptake–driven adipogenesis mechanism, which occurs in addition to the hyperplastic expansion (increased adipocyte number) of PFs in OAT in GO (11). In particular, lower lipolytic activity with similar (low) FA synthesis accompanied by increased expression of a depot-specific FA transporter (*SLC27A6*) were observed in OAT from healthy individuals (OAT-H) and GO patients (OAT-GO) compared with WAT (11).

Involvement of mitochondrial dysfunction in the orbital fat expansion in GO is suggested by increased expression of the uncoupling protein *UCP1* in OAT from GO. This has been observed both in human models—ex vivo analysis of OAT-GO (11), GO-targeted PFs by *TSHR* activation (16, 17)—and a mouse model of *TSHR*-induced GO (18). *UCP1* expression in mitochondria is a known feature of BAT, which dissipates energy as heat by uncoupling mitochondrial oxidative-phosphorylation (OXPHOS) from adenosine 5′-triphosphate (ATP) production and also plays an important role in mitochondrial function (19). Hyperplastic expansion of adipocytes in human BAT caused by mitochondrial dysfunction via mutation of *MFN2* has recently been reported (20). Substantially increased expression of *MFN2* has also been observed in OAT from GO patients compared with OAT-H in our recent study (11). Overexpression of adiponectin, which is also a mitochondrial function modulator, has been shown to induce expansion both of OAT and BAT in a mouse model (21, 22).

These factors, together with the identified specific molecular signatures of OAT (eg, *Sirtuin*/*Wnt*/*Ca *+ signaling pathways) from our recent study suggest a role for mitochondria in the development of GO (11). Apart from being a cellular “powerhouse,” mitochondrial OXPHOS and its biosubstrates, such as ATP/adenosine 5′-diphosphate (ADP) and guanosine 5′-triphosphate/guanosine 5′-diphosphate (GTP/GDP), play fundamental roles in the regulation of cell metabolism through interacting with complex molecular cascades, for example, cell proliferation and differentiation (23, 24).

Our present study investigated the hypothesis that dysfunction of mitochondria plays a role in the FA-uptake–driven adipogenesis in OAT expansion in GO. Our investigation demonstrated mitochondrial dysfunction in PFs from OAT-GO, which linked with the confirmed FA-uptake–driven adipogenesis from in vitro adipogenesis analysis.

## Materials and Methods

All reagents were obtained from Sigma-Aldrich and tissue culture components from Cambrex unless otherwise stated.

### Adipose Tissue Collection and Preparation

Adipose tissue was collected with informed written consent and local research ethics committee approval. WAT (subcutaneous) was from 10 patients undergoing elective open abdominal or breast surgery for nonmetabolic conditions. OAT from GO patients (n = 13) were from 10 inactive GO patients with a clinical activity score of less than 2, 3 active GO patients with a clinical activity score of 3 or greater undergoing 2-wall or 3-wall orbital decompression surgery. Most of the GO patients had carbimazole treatment, radioimmunoassay, and/or thyroidectomy in the past; 2 GO patients received no antithyroid treatment; and 2 were receiving carbimazole treatment while the OAT samples were obtained. OATs from non-GO patients (n = 11) who were free of thyroid or other inflammatory eye disease and were undergoing augmented upper eyelid blepharoplasty surgery. OAT-PFs were obtained from adipose tissue explants and WAT-PFs were obtained by collagenase digest, both as previously described (17). Cells were used at low passage number (< 5), hence not all samples were analyzed in all experiments.

### Preadipocyte/Fibroblast Culture and In Vitro Adipogenesis

PFs were cultured in Dulbecco’s modified Eagle’s medium/F12 10% flow cytometry (FACS) (complete medium; CM). Adipogenesis was induced in confluent cells by replacing with adipogenic medium (ADM) containing 10% fetal calf serum, biotin (33 μM), panthothenate (17 μM), 3,5,3′-triiodothyronine (1 nM), dexamethasone (100 nM), thiazolidinedione (1 μM), and insulin (500 nM) for 17 days. Adipogenesis was assessed by microscopic examination to detect the characteristic morphological changes (cell rounding, accumulation of lipid droplets), acquisition of lipid-filled droplets (oil-red-O staining), and transcript measurement of terminal adipogenic marker (lipoprotein lipase) by quantitative polymerase chain reaction (qPCR) as described previously (17).

For experiments using exogenous fatty acid (FA), a fatty acid mixture (200 µM) comprising oleate:palmitate:linoleate (45:30:25%) bound to bovine serum albumin was added to CM throughout the whole time culture as previously described (25), with/without ADP, GDP, oligomycin (mitochondrial inhibitor), or adenosine analog (AICAR) for 17 to 19 days. Adipogenesis was analyzed by foci of differentiation (groups of cells with lipid droplets), which were counted in ten different fields for each experimental condition as described before (16).

### Triglyceride Extraction From Differentiated Preadipocytes/Fibroblasts and ILAB Analysis

Confluent PFs were cultured in ADM with and without the supplementation of FA, oleate:palmitate:linoleate (45:30:25%), for 10 days in a 24-well plate. Cellular TAG from the differentiated PFs were analyzed as previously described (25). In brief, cell lysates were obtained using lysis buffer (1% IGEPAL CA-630, 150-mM NaCl, and 50-mM Tris-HCl [pH8.0]) and sonication. Some of the lysates were used for protein quantification using BCA assay (Bio-Rad, DC protein assay kit). The lysates used for TAG analysis were heated at 95 °C for 30 minutes and centrifuged at 12 000*g* for 10 minutes after cooling. Cellular TAG concentration was measured using an enzymatic assay (TAG assay, Instrumentation Laboratory) with glycerol standards, and run on an ILAB 650 clinical analyzer (Instrumentation Laboratory). The normalized TAG content per unit protein was obtained using the following calculation: TAG (µM) ÷ protein (mg/mL).

### NAD(P)H and Adenosine 5′-Triphosphate Measurement

Confluent PFs were cultured in CM or ADM for 4 days and changed to CM before the following experiments. Cell number was counted using Cellometer from Nexcelom. MTS assay was performed (indicating the production of NAD(P)H (26)) using CellTiter 96 AQueous One Solution Assay from Promega according to the manufacturer’s instructions, 490-nm absorbance was measured after 2 hours’ incubation and normalized by cell number. Cells were harvested after culture in CM or ADM for 4 days, and total cellular ATP (µM) was measured using standard ATP dilutions by luminescent ATP detection assay kit from Abcam according to the manufacturer’s instructions. Each condition had 4 repeats for the above experiments.

### Mitochondria Number Analysis by Relative Quantitative Polymerase Chain Reaction

DNA was extracted from confluent PFs from OAT-H, OAT-GO, or WAT using standard protocol, and qPCR was conducted using SYBR Green incorporation measured on a Stratagene MX 3000 as previously described (16). Comparative 1PCR was measured and expressed relative to a reference DNA *RPL13A* for mitochondria DNA cytochrome b (*Cytob*) detection to determine relative mitochondria number using the primers as follows: *RPL13A*, forward 5′- CTCAAGGTCGTGCGTCTG-3′ and reverse 5′-TGGCTTTCTCTTTCCTCTTCT-3′; *Cytob*, forward 5′-GCGTCCTTGCCCTATTACTATC-3′, and reverse 5′-CTTACTGGTTGTCCTCCGATTC-3′ as described previously (27).

### Flow Cytometry Analysis

Confluent PFs were cultured in CM (as day 0), and changed to CM or ADM for 4 days. Cell numbers were counted by Cellometer from Nexcelom, and followed the procedures using FACS (BD FACSCanto II systems) with FACSDiva 6.0 software from Becton Dickinson and Co as described previously (28). In brief, cells were fixed with 100% methanol and permeabilized with 0.1% phosphate-buffered saline–Tween20 for 20 minutes. Cells were then incubated with 10% normal goat serum/0.3-M glycine to block nonspecific protein-protein interactions followed by primary antibody of mitochondrial-cytochrome-oxidase (MtCO_2_) (ab3298 from Abcam [RRID: AB_303683, https://antibodyregistry.org/search.php?q=AB_303683], 1 µg/L × 10^6^ cells) or isotype control antibody (mouse immunoglobulin G) for 30 minutes. The secondary antibody used Alexa Fluor 488 antimouse immunoglobulin G (1:500 dilution) for 30 minutes, and then fluorescence emissions were collected for 10 000 cells by FACS analysis. FACS intensity of positive MtCO_2_ staining referenced to negative control was analyzed using FlowJo software version 10.0.5 (Tree Star Inc).

### Mitochondrial Functional Assays by Seahorse XFe96 Analyzer

Basal Assay medium and XFe96 consumables were purchased from Agilent Technologies; Draq5 was purchased from Abcam for DNA staining. Oxygen consumption rate (OCR) and extracellular acidification rate (ECAR) were measured with the Seahorse XFe96 (Agilent Technologies) using Mito Stress kit according to the manufacturer instructions as described previously (29). Briefly, cells were seeded at 1 × 10^4^ per well on a Seahorse plate. At 48 hours post seeding, confluent PFs were then transferred into nonbuffered Seahorse assay medium containing 17.5-mM glucose, 1.5-mM sodium pyruvate, and 2.5 mM of L-glutamine with 3% fetal calf serum. Basal cellular respiration rate was first measured followed by oligomycin A injection (1 µM) to inhibit ATP synthase. Maximal respiration capacity was determined in 3.5-µM FCCP. Finally, nonmitochondrial respiration rate was determined using a combination of rotenone (0.5 µM) and antimycin A (1 µM). Measurements were performed with 3 cycles including 4 minutes of medium mixing followed by 3 minutes of measurements. For data normalization, cells were fixed for 20 minutes with 4% paraformaldehyde. Cell nuclei were then stained for 1 hour with Draq5 (ab108410 from Abcam) 1:10000 diluted in phosphate-buffered saline + 0.1% Tween-20. Stained cells were then detected with an Odyssey Scanner (Li-Cor). Fluorescent intensity per well was used to normalize respiration values per well. The average of the nonmitochondrial respiration measures was then subtracted from each corresponding condition/time point per well; all similar condition measurements per well were then averaged. On day 0 (48 hours post seeding), PFs from OAT, OAT-GO, and WAT (n = 4 for each) in CM were measured with 5 repeats of each condition from one 96-well plate; day 4 (or day 7) PFs (n = 4) in CM and ADM were measured from two 96-well plates. PFs from WAT (n = 4), OAT (n = 8) and OAT-GO (n = 9) were used, and 2 sets of independent experiments were performed for OAT (n = 4 and 4) and OAT-GO (n = 4 and 5), respectively.

Cellular ATP production rates by mitochondrial OXPHOS and glycolysis were calculated using obtained OCR and ECAR from Seahorse analysis, taking into account the acidification rates due to mitochondrial CO_2_ production as described previously (30, 31).

### Statistical Analyses

Results were analyzed using Prism 5 (version 5.02), and data normality was initially analyzed using the Kolmogorov-Smirnov test. To compare groups we used the *t* test for normally distributed variables and the Mann-Whitney test for nonnormally distributed data. Differences between groups were analyzed using one way analysis of variance. We applied Dunnett’s multiple comparison post hoc test for multiple comparisons when identifying statistically significant differences. In all cases *P* less than .05 was considered significant. Data are presented as mean ± SEM.

For correlation analysis, the percentage increased FA-uptake adipogenesis by GDP and measured OXPHOS-ATP/glycolysis-ATP of the cells were analyzed for normality using the Kolmogorov-Smirnov test. All the data were normally distributed and correlation was analyzed using the Pearson correlation.

## Result

### Depot-specific Mitochondrial Adenosine 5′-Triphosphate Production by Preadipocytes/Fibroblasts From Orbital Adipose Tissue in Early Adipogenesis

We induced in vitro adipogenesis using ADM with a mixed hormonal cocktail in normal CM on human primary PFs for 17 days. The differentiated cells displayed cell rounding and accumulation of lipid droplets (TAG formation) with positive oil-red-O staining and the induction of lipoprotein lipase expression (a marker of late adipogenesis) as previously reported (17).

**Figure 1.**
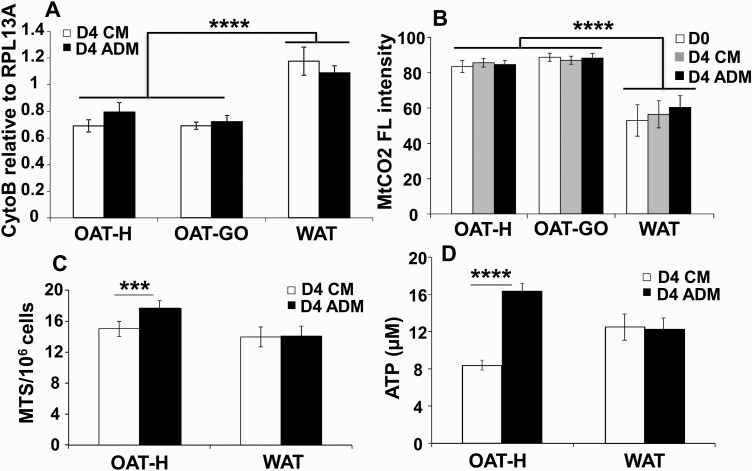
Mitochondria analysis and cellular adenosine 5′-triphosphate (ATP) production of orbital adipose tissue preadipocytes/fibroblasts (OAT-PFs) in adipogenic-medium (ADM)-adipogenesis compared with white adipose tissue (WAT). Confluent PFs from healthy OAT (OAT-H), Graves orbitopathy (OAT-GO), or WAT were cultured in complete medium (CM) or ADM for 4 days. A, DNA extracted from OAT (n = 3) and OAT-GO (n = 3) or WAT-PFs (n = 4) on day 4, relative quantitative polymerase chain reaction of cytochrome b (*Cytob*, mitochondrial DNA) to *RPL13A* (reference genomic DNA) was performed. B, Mitochondrial-cytochrome-oxidase (MtCO_2_) antibody was analyzed by flow cytometry from confluent OAT (n = 4), OAT-GO (n = 4), and WAT-PFs (n = 7) at day 0 in CM or day 4 in CM and ADM, the percentage of flow cytometric (FL) intensity, positive mitochondrial-cytochrome-oxidase (MtCO_2_) staining, is shown referenced to negative control. C, MTS assay (indicating the production of NAD(P)H and mitochondrial oxidative-phosphorylation (OXPHOS) activity (26)) was performed on PFs from OAT-H (n = 3) and WAT-PFs (n = 4) on day 4 in CM and ADM, 490 nm absorbance normalized by cell number. D, Total level of cellular ATP (luminescent ATP detection assay kit) was measured from OAT-H (n = 7) and WAT-PFs (n = 4) on day 4 in CM and ADM. Histograms = mean ± SEM of all samples studied. *T* test was used for statistical analysis. ***P less than or equal to .001; ****P less than or equal to .0005.

We compared the early stages of adipogenesis, before the formation of adipocytes, in confluent PFs from WAT, OAT-H, or OAT-GO cultured in CM (nondifferentiating) or ADM (differentiating) conditions. We analyzed mitochondria numbers by relative qPCR of mitochondrial DNA, *Cytob*, to a reference genomic DNA, *RPL13A*. Fig. 1A demonstrates a lower (35.3 ± 3.2%) mitochondrial number in OAT-H or OAT-GO, either in CM or ADM condition on day 4 compared to PFs from WAT. Interestingly, MtCO_2_ levels were significantly higher (52.8 ± 2.5%) in OAT-H and OAT-GO compared with PFs from WAT using FACS (Fig. 1B). MtCO_2_ is a necessary component of the respiratory chain of mitochondria for OXPHOS-ATP production (32).

Furthermore, we detected a substantially higher production of NAD(P)H (1.23 ± 0.06-fold increase), indicating increased mitochondrial OXPHOS activity (MTS assay/10^6^ cells; Fig. 1C) (26) and increased total cellular ATP (1.97 ± 0.03-fold increase) of differentiating PFs from OAT-H cultured in ADM compared with CM condition (nondifferentiating PFs) (Fig. 1D), which was not observed in WAT. The increased production of NAD(P)H and total cellular ATP were also observed in differentiating PFs from OAT-GO compared with nondifferentiating-PFs (Supplementary Fig. S1) (33); however, significantly lower fold increases were observed when compared with OAT-H. There was no significant difference in cell number between PFs cultured in CM and ADM on day 4 (Supplementary Fig. S2) (33).

### Dysfunction of Live Adenosine 5′-Triphosphate Production via Mitochondrial Oxidative-Phosphorylation in Preadipocytes/Fibroblasts From Graves Orbitopathy Orbital Adipose Tissue

We next explored live ATP production rates by mitochondrial OXPHOS or cellular glycolysis according to OCR and ECAR measured by 96-well Seahorse analyzer, as previously described (30, 31). Analyses were performed at basal day 0 in CM condition or days 4 and 7 in CM and ADM conditions at early adipogenesis, that is, before observation of any differentiated adipocytes. All the obtained data were referenced to cell DNA staining at each time point and condition.

**Figure 2.**
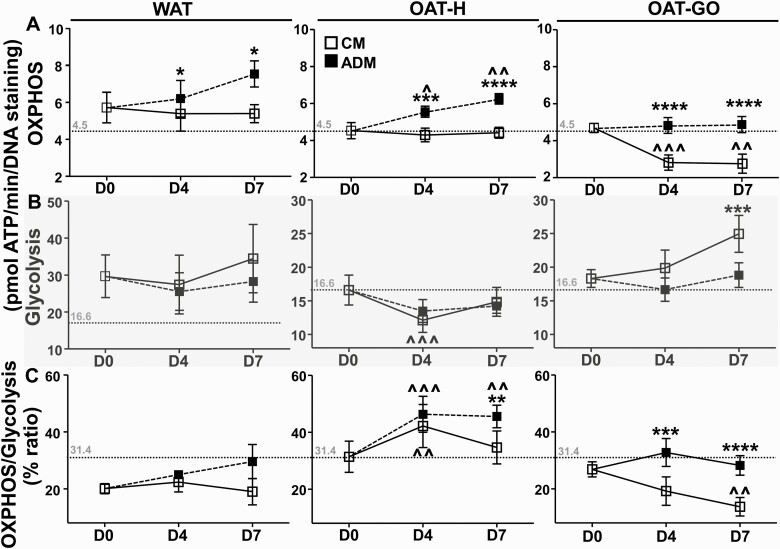
Live adenosine 5′-triphosphate (ATP) production from oxidative-phosphorylation (OXPHOS)/glycolysis in differentiating or nondifferentiating preadipocytes/fibroblasts (PFs) from white adipose tissue (WAT), healthy orbital adipose tissue (OAT-H), and Graves orbitopathy OAT (OAT-GO). Confluent PFs were cultured in 96-well plates, oxygen consumption rate (OCR) as OXPHOS-ATP and extracellular acidification rate (ECAR) as glycolysis-ATP were measured using Mito Stress kit from WAT (n = 4), OAT (n = 8), and OAT-GO (n = 9) using Seahorse analyzer. A, OXPHOS-ATP, and B, glycolysis-ATP were measured as pmol ATP/min on day (D)0 plate in complete culture medium (CM, white square), or after PFs in CM (nondifferentiating) or adipogenic medium (ADM; black square) (differentiating PFs) for 4 or 7 days and referenced to DNA staining. C, Percentage ratio of OXPHOS vs glycolysis on D0, 4, and 7 with CM or ADM condition were presented. Basal day 0 levels of ATP production by OXPHOS (4.5 pmol ATP/min), glycolysis (16.6 pmol ATP/min), and its ratio (31.4%) of PFs from OAT-H are displayed and indicated by dash lines. Histograms = mean ± SEM of all samples studied. Data were normally distributed, a one-way analysis of variance with the Dunnett multiple comparison test or *t *test was used to compare D4 or D7 time points with D0 (^); or *t* test was used to compared between CM and ADM on D4 and D7 (*). *P less than .05; **P less than or equal to .01; ***P less than or equal to .005; ****P less than or equal to .0005.

During early adipogenesis, an increased OXPHOS ATP level of differentiating PFs was observed compared to nondifferentiating PFs on day 4 from WAT, OAT-H, and OAT-GO, and further increased day 7 from WAT and OAT-H, but not OAT-GO (Fig. 2A). We then looked in detail into changes in OXPHOS and glycolysis ATP levels in PFs from OAT-H and OAT-GO separately.

From OAT-H, nondifferentiating-PFs had substantially decreased glycolysis ATP levels on day 4 compared with days 0 and day 7 (Fig. 2B), accompanied by unchanged levels of OXHPOS-ATP (see Fig. 2A). Consequently, there was a higher OXPHOS-ATP/glycolysis-ATP ratio on day 4 compared with days 0 and 7 in nondifferentiating PFs (Fig. 2C). Interestingly, in differentiating PFs (through adipogenesis), significantly higher levels (28.8 ± 11.7%; 46.9 ± 15.3%) of OXPHOS-ATP production (see Fig. 2A) and OXHPOS-ATP/glycolysis-ATP ratio (56 ± 10.8%; 68.9 ± 23%) (see Fig. 2C) with unchanged glycolysis ATP (Fig. 2B) were observed on day 4 or 7 when compared with day 0, respectively.

From OAT-GO, nondifferentiating PFs showed a trend to increased glycolysis-ATP levels (see Fig. 2B) contrasting with a sharp and consistent drop (39.7 ± 7.5%; 40.8 ± 10.7%) of OXPHOS-ATP levels (see Fig. 2A) on day 4 or 7 when compared with day 0, respectively. Consequently, there was a trend to decreased ratio of OXPHOS-ATP/glycolysis-ATP on days 4 and 7 compared with day 0 in nondifferentiating PFs (see Fig. 2C). Through adipogenesis (differentiating PFs) in OAT-GO, unchanged levels of OXHPOS-ATP/glycolysis-ATP and its ratio on days 0, 4, and 7 were observed, contrasting with our findings in OAT-H (see Fig. 2).

In summary, there is a reduction in ATP production by OXPHOS in OAT-GO nondifferentiating-PFs that recovers somewhat in differentiating PFs. By contrast, this reduction of OXPHOS-ATP production is absent in OAT-H nondifferentiating PFs, and levels increase as PFs differentiate.

### Fatty Acid–Uptake–driven Adipogenesis and the Link With Mitochondrial in Orbital Adipose Tissue

Cells with lipid droplets and positive oil-red-O staining were identified as differentiated adipocytes, and a foci count was performed as described previously (16).

**Figure 3.**
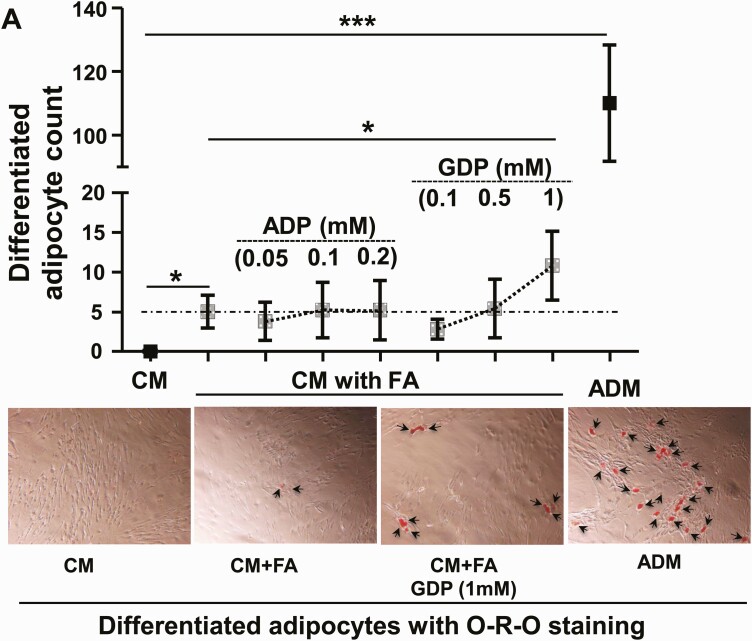

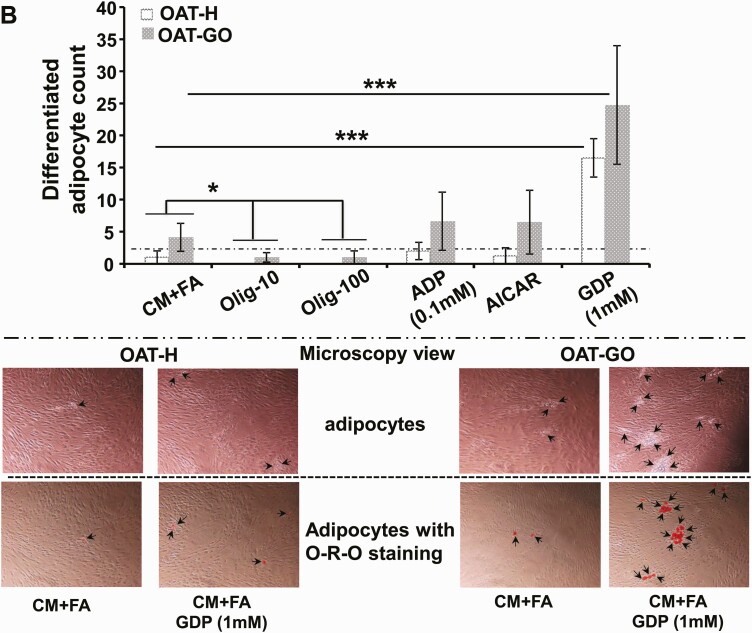
The link of mitochondrial oxidative-phosphorylation (OXPHOS) in fatty acid (FA)-uptake–driven adipogenesis of preadipocytes/fibroblasts (PFs) from orbital adipose tissue (OAT). A, Confluent cells from healthy OAT (OAT-H) (n = 1) and Graves orbitopathy OAT (OAT-GO) (n = 4), passages 2 to 4, were cultured in complete culture medium (CM) or adipogenic medium (ADM, black square), or with FA supplement in CM (square with dot) for 17 days with treatment of adenosine 5′-diphosphate (ADP) (0.05 mM, 0.1 mM, or 0.2 mM), or guanosine 5′-diphosphate (GDP, 0.1 mM, 0.5 mM, or 1 mM). B, Confluent cells from OAT-H (n = 4, white column) or OAT-GO (n = 8, black dot column), passages 2 to 3 were cultured in CM with FA supplement for 19 days, with/without 10-ng/mL oligomycin-A (Olig-10), 100-ng/mL oligomycin-A (Olig-100), 0.1-mM ADP, 0.1-mM adenosine analogue (AICAR), and 1-mM GDP. Cells were then fixed and stained using oil-red-O technique. Differentiated adipocytes (with lipid droplets) were observed and counted in 10 different fields. Representative photos are shown with arrows indicating differentiation adipocytes (×10 magnification). Histograms = mean ± SEM of all samples studied. Normal (*t* test) or nonnormal (Mann-Whitney test) distributed data were analyzed accordingly. *P less than .05; ***P less than .005.

To investigate FA-uptake–driven adipogenesis in vitro, we cultured confluent PFs from OAT-H or OAT-GO with extra free FA using a mixture of 3 major TAG FA, oleate:palmitate:linoleate (45:30:25%) as described in the investigation of FA uptake in PFs from WAT (25). As expected, differentiated adipocytes were apparent in ADM after 17 days of full-differentiation protocol with an adipogenesis hormonal cocktail, whereas no adipogenesis was observed in the CM condition (Fig. 3A). The addition of the FA supplement to ADM led to the differentiated adipocytes as early as day 10, which had about 3-fold higher cellular TAG per protein unit compared with adipocytes in ADM alone, which were still at the early-middle stage of adipogenesis (Supplementary Fig. S3) (33).

The effect of FA and the role of mitochondria in OAT adipogenesis were further investigated by culturing confluent PFs from OAT-H and OAT-GO with FA supplement in CM without the adipogenesis hormonal cocktail and with the addition of different concentrations of mitochondrial biosubstrates, ADP (0.05, 0.1, 0.2 mM) and GDP (0.1, 0.5, 1 mM) for 17 days. To our surprise, we observed differentiated adipocytes by feeding FA only in CM for 17 days both to OAT-H and OAT-GO PFs (OAT-H n = 1 and OAT-GO n = 4; see Fig. 3A). This type of FA-uptake–driven adipogenesis was substantially enhanced by 1-mM GDP in CM reaching to 7% to 18% of ADM hormonal cocktail–induced adipogenesis (see Fig. 3A). In contrast, no significant changes in the FA-uptake–driven adipogenesis were observed with the ADP supplement in PFs both from OAT-H and OAT-GO (see Fig. 3A).

We then analyzed the effect of the optimal 1-mM concentration of GDP and FA supplement in FA-uptake–driven adipogenesis in additional samples. We also tested the effect of ADP and AICAR/oligomycin, respectively). The results are summarized in Fig. 3B; we observed differentiated adipocytes from OAT-H (n = 1 out of 4) and OAT-GO PFs (n = 7 out of 8) by feeding FAs in CM. Furthermore, substantially increased FA-uptake–driven adipogenesis in PFs from all OAT-H and OAT-GO was observed on the addition of 1-mM GDP (see Fig. 3B). In contrast, the addition of AICAR or ADP had no effect on FA-uptake–driven adipogenesis, and the use of different concentrations of the mitochondrial inhibitor oligomycin abolished the induced FA-uptake–driven adipogenesis of all PFs from OAT-H and OAT-GO (see Fig. 3B).

We replicated the effects of GDP and ADP with FA supplement in CM on OAT-PFs in further experiments (Supplementary Fig. S4) (33).

In summary, FA-triggered adipogenesis was observed in OAT-H PFs from 4 out of 5 individuals and 8 out of 10 OAT-GO PFs samples from GO patients. In all cases, this was substantially enhanced by the addition of GDP supplement.

### Correlation of Fatty Acid–Uptake–driven Adipogenesis by Guanosine 5′-Diphosphate With Increased Oxidative-Phosphorylation/Glycolysis Capacity of Preadipocytes/Fibroblasts From Graves Orbitopathy Orbital Adipose Tissue

**Figure 4.**
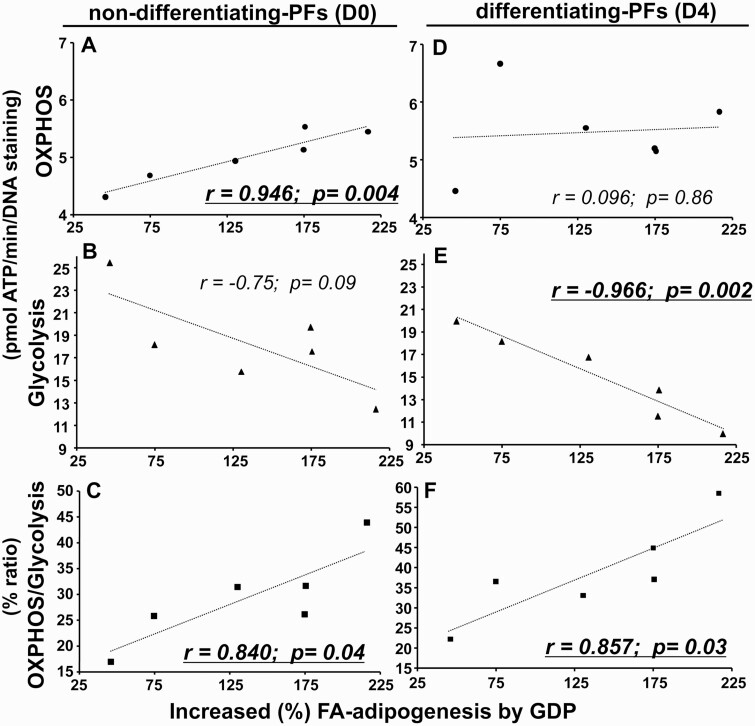
Correlations of fatty acid (FA)-uptake–driven adipogenesis by guanosine 5′-diphosphate (GDP) treatment with levels of oxidative-phosphorylation (OXPHOS)/glycolysis–adenosine 5′-triphosphate (ATP) in preadipocytes/fibroblasts (PFs) from Graves orbitopathy orbital adipose tissue (OAT-GO). The scatterplot showing the relationship between percentage induced FA-uptake–driven adipogenesis by GDP + FA vs FA only, and levels of A, OXPHOS-ATP (dot); B, glycolysis-ATP (triangle); and C, the percentage ratio of OXPHOS/glycolysis (square) in nondifferentiating-PFs at basal day 0; or D, OXPHOS-ATP levels; E, glycolysis-ATP levels; and F, the percentage ratio of OXPHOS/glycolysis of differentiating PFs on day 4 adipogenesis. Statistically significant Pearson correlations for normally distributed data are underlined.

In 6 OAT-GO samples we had data both from Seahorse analysis (see Fig. 2) and FA-induced adipogenesis (see Fig. 3). The further enhanced FA-uptake–driven adipogenesis by GDP treatment (percentage increase vs FA feeding only) significantly and positively correlated with the measured OXPHOS-ATP levels (*r* = 0.946, *P* = .004) (Fig. 4A), no significant correlation with the levels of glycolysis-ATP (*r* = –0.75, *P* = .09) (Fig. 4B), and significantly and positively correlated with the OXPHOS-ATP/glycolysis-ATP ratio (*r* = 0.840, *P* = .04) (Fig. 4C) in nondifferentiating PFs at basal day 0. The further-enhanced FA-uptake–driven adipogenesis by GDP treatment had no significant correlation with the measured levels of OXPHOS-ATP (*r* = 0.096, *P* = .86) (Fig. 4D), but significantly correlated negatively with the levels of glycolysis-ATP (*r* = –0.966, *P* = .002) (Fig. 4E) and positively with OXPHOS-ATP/glycolysis-ATP ratio (*r* = 0.857, *P* = .03) (Fig. 4F) in differentiating PFs on day 4.

## Discussion

Our study demonstrated increased cellular ATP production via mitochondrial OXPHOS during adipogenesis of PFs from OAT but not WAT. Mitochondrial dysfunction was observed with disrupted levels of OXPHOS-ATP/glycolysis-ATP and its ratio in PFs from OAT-GO compared with OAT-H. Furthermore, FA supplementation was able to trigger in vitro adipogenesis in PFs both from OAT-H and OAT-GO, which was substantially enhanced by addition of GDP and diminished by mitochondrial inhibitor. The enhanced FA-uptake–driven adipogenesis by GDP was significantly and positively correlated with ratios of OXPHOS-ATP/glycolysis-ATP at basal nondifferentiating PFs or early adipogenesis of differentiating PFs from OAT-GO. Taken together, these observations suggest an essential role of mitochondrial OXPHOS-ATP production and its biosubstrate GDP in the depot-specific FA-uptake–driven adipogenesis of OAT-H and one exacerbated in GO by mitochondrial dysfunction.

Our investigation employed a well-established cell model, a heterogeneous population (PFs) derived from the entire stromal vascular fraction of human OAT and WAT (10, 34). The key findings of this study were confirmed by using more than one independent technique and the observations from our recent ex vivo OAT analysis (11) as discussed later. The low availability of human orbital PFs precludes the use of a model comprising a homogeneous cell type, although this would be preferable.

We and others have described the multidifferentiation MSC potential of adipose tissue–derived PFs (9-11, 35). Classically, in vitro adipogenesis is triggered by ADM with a mixed hormonal cocktail to activate key transcriptional factors, such as *PPAR* and *CEBP*, (36, 37), as routinely used in our laboratory (17). Our present study demonstrated that FAs alone were able to induce in vitro adipogenesis in PFs from OAT-H and OAT-GO. This confirms the depot-specific FA-uptake–driven adipogenesis in OAT, which was suggested by ex vivo analysis of human adipose tissue from our recent study (11). Furthermore, we previously demonstrated an abundant expression of the FA transporter, *SLC27A6*, with limited FA synthesis/lipolysis in OAT in GO supporting the excessive FA-uptake–driven adipogenesis in GO (11). Thyroid hormone, *PPAR*γ ligand, and cytokines (eg, tumor necrosis factor or interleukin 6) play important roles in the pathogenesis of GO, as reviewed in (2), which may also contribute to OAT adipogenesis through their role in the regulation of lipid metabolism via FA transporter (38, 39). Further investigation is needed to dissect the important role of the FA transporter system, in the specific context of the MSC phenotype of OAT-PFs, in the FA-uptake–driven adipogenesis in GO (9-11).

Our study revealed that inhibition of mitochondrial OXPHOS by oligomycin abolished the FA-uptake–driven adipogenesis of OAT, which supports the important function of mitochondria in adipogenesis as reported in other fat depots (20, 40). However, AICAR or ADP, the activator of adenosine 5′-monophosphate kinase, had no effect on the FA-uptake–driven adipogenesis of OAT, thereby eliminating the adenosine 5′-monophosphate kinase pathway, which plays important roles in other fat depots (41). By contrast, our present study demonstrated that the depot-specific FA-uptake–driven adipogenesis was substantially enhanced by supplementing GDP in PFs both from OAT-H and OAT-GO. The important role of GDP in mitochondrial function and cellular metabolism has been reported in OXPHOS-ATP production (42) or via GTP/GDP exchange (43). Our study suggests that the reduced mitochondrial OXPHOS results in more available GDP to form a feedback loop to regulate the FA-uptake–driven adipogenesis in OAT.

The present study demonstrated that mitochondrial function in OAT-PFs during adipogenesis is linked with an inducible total cellular ATP production via mitochondrial OXPHOS. It contrasts with WAT-PFs, or other human stem cells, that have unchanged or even decreased levels of total cellular ATP, apart from increased OXPHOS-ATP through adipogenesis as observed in this study and by others (40, 44). Our investigation observed a depot-specific higher level of mitochondrial cytochrome-C oxidase (32) with a low mitochondria number in PFs from OAT compared with WAT, suggesting increased mitochondrial activity rather than numbers. Furthermore, in OAT-H, early adipogenesis (days 0, 4, and 7) is accompanied by a U-shaped distribution (high, low, high) of glycolysis-ATP and unchanged OXPHOS-ATP levels. In contrast, levels of OXPHOS-ATP fell sharply with compensated high level of glycolysis-ATP from nondifferentiating-PFs from OAT-GO, indicating sustained mitochondrial dysfunction of PFs in GO.

Our recent study highlights the key role of enhanced proliferation of PFs synergized with FA-uptake–driven adipogenesis in the hyperplastic expansion of OAT in GO (11). Studies have shown that the Warburg phenotype of proliferating cells is important to have enhanced glycolysis with suppressed mitochondrial-OXPHOS; however, nonproliferating cells display higher OXHPOS-ATP production with inhibited glycolysis (45, 46). Our data clearly demonstrate OXPHOS-ATP levels falling sharply with a compensated high level of glycolysis-ATP production in nondifferentiating-PFs from OAT-GO, which may trigger the proliferation of PFs in GO (45, 46). Once proliferation is induced, we see higher levels of OXPHOS-ATP and low levels of glycolysis-ATP through adipogenesis in differentiating PFs from OAT-H reported here, which may in turn lead to inhibition of proliferation and drive PFs to differentiate (45, 46). This study provides further support of the positive correlation noted between FA-uptake–driven adipogenesis by GDP and the ratios of OXPHOS-ATP/glycolysis-ATP through adipogenesis of PFs from OAT-GO. We hypothesize that the levels of mitochondrial OXPHOS-ATP and metabolites (GDP) play central roles in triggering PF proliferation and excessive FA-uptake–driven adipogenesis of OAT in GO. The uncorrected dysfunction of mitochondria in PFs from OAT-GO leads to maintained levels of low-OXPHOS/high-glycolysis causing excessive proliferation, and more available GDP promoting FA-uptake–driven adipogenesis in OAT expansion in GO.

**Figure 5.**
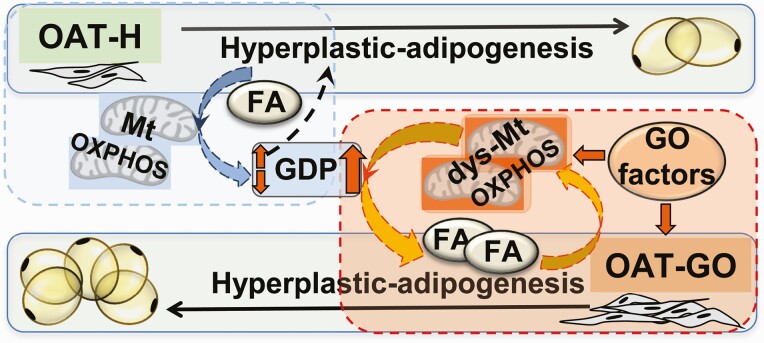
The essential role of mitochondrial oxidative-phosphorylation (OXPHOS) for fatty acid (FA)-uptake–driven adipogenesis in the hyperplastic orbital adipose tissue (OAT) expansion. Our study demonstrated a depot-specific cellular adenosine 5′-triphosphate (ATP) production via mitochondrial-OXPHOS through adipogenesis in OAT preadipocytes/fibroblasts (PFs). Dysfunction of mitochondria (dys-Mt) with disrupted OXPHOS-ATP production was observed in PFs from OAT–Graves orbitopathy (GO) due to GO factors, such as thyrotropin receptor/insulin-like growth factor 1 receptor (*TSHR/IGF1R*), etc (11, 17, 18, 21, 53-55). Hypothesis: Mitochondrial OXPHOS-ATP production through adipogenesis is important in maintaining OAT stability by forming a beneficial relationship with the available guanosine 5′-diphosphate (GDP) in PFs to maintain a healthy (low) level of proliferation/adipogenesis in healthy (OAT-H, highlighted in dashed blue-square). The pathological fall in OXPHOS-ATP with compensated high level of glycolysis-ATP by GO factors triggered the proliferation of PFs in OAT in GO, and kept low-level mitochondrial-OXPHOS and consequently higher GDP availability driving excessive FA-uptake–driven adipogenesis in OAT in GO (highlighted in red square). Dashed lines indicate proposed mechanism.

The OAT depot–specific cell signaling cascades (13, 14) play a central role in the pathogenesis of OAT expansion in GO through the interplay of *TSHR*/*PKA*, *IGF1R*/*PI3K*/*Akt*, *mTORC1*, and the downstream nucleus factor, *FOXOs*, (7, 47-50), which are also important in mitochondrial function (43). More work is needed to clarify how *TSHR*/*IGF1R* interfere with mitochondria via GDP and OXPHOS-ATP production in triggering proliferation and FA-uptake–driven adipogenesis of OAT, for example, via pathological activation of *UCP-1* (11), or interference with the adenylate cyclase system of GTP/GDP exchange to allow the activation of GO targeted *TSHR*/*IGF1R* (51, 52). However, we hypothesize that the resultant mitochondrial dysfunction in association with the FA-uptake mechanism drives OAT expansion in GO (summarized in Fig. 5).

## Data Availability

All data generated or analyzed during this study are included in this published article or in the data repositories listed in “References.”

## Abbreviations

ADP: adenosine 5′-diphosphate
AICAR: adenosine analogue
ATP: adenosine 5′-triphosphate
BAT: brown adipose tissue
CM: complete medium
ECAR: extracellular acidification rate
FA: fatty acid
FACS: flow cytometry
GDP: guanosine 5′-diphosphate
GO: Graves orbitopathy
IGF1R: insulin-like growth factor 1 receptor
MSC: mesenchymal stem cell
OAT: orbital adipose tissue
OAT-GO: orbital adipose tissue from Graves orbitopathy patients
OAT-H: orbital adipose tissue from healthy individuals
OCR: oxygen consumption rate
OXPHOS: oxidative-phosphorylation
PFs: preadipocytes/fibroblasts
qPCR: quantitative polymerase chain reaction
TAG: triglyceride
TSH: thyrotropin
TSHR: thyrotropin receptor
WAT: white adipose tissue

## Additional Information

***Disclosures:*** The authors have nothing to disclose.
